# The more critical murderer of atherosclerosis than lipid metabolism: chronic stress

**DOI:** 10.1186/s12944-018-0795-4

**Published:** 2018-06-19

**Authors:** Ling-bing Meng, Ruomei Qi, Lei Xu, Yuhui Chen, Zemou Yu, Peng Guo, Tao Gong

**Affiliations:** 1 Neurology Department, Beijing Hospital, National Center of Gerontology, No.1 Dahua Road, Dong Dan, Beijing, 100730 People’s Republic of China; 2 The MOH Key Laboratory of Geriatrics, Beijing Hospital, National Center of Gerontology, No.1 Dahua Road, Dong Dan, Beijing, 100730 People’s Republic of China; 3 Department of Neurology, Peking University First Hospital, Beijing, People’s Republic of China; 4 Department of Orthopedics, The Fourth Hospital of Hebei Medical University, Shijiazhuang, China

**Keywords:** Cerebrovascular disease, Atherosclerosis, Vascular intimal thickness, Vascular media thickness, Chronic stress, Low density lipoprotein cholesterol, Total cholesterol

## Abstract

**Background:**

The mortality of atherosclerotic cerebrovascular disease is on the rise, and changes in intimal and media thickness are a leading cause of cerebral ischemia-related death. Levels of low density lipoprotein cholesterol (LDLC), total cholesterol (TC), and chronic stress (CS) are all recognized risk factors for atherosclerosis (AS). However, the leading independent risk factor is indistinct. This study explored the effects of chronic stress, LDLC, and TC on AS and intimal and media thickness, preliminarily explored the main risk factor of AS, and analyzed the related histocyte mechanisms for macrophages and endothelial cells.

**Methods:**

Conditions include normal, high-fat diet (HF), and HF plus CS. The correlations between intimal and media thickness and general risk factors were analyzed using χ2, Spearman’s rho test, and multiple linear regression. Univariate Cox regression was used to identify potential factors that affect the non-depression time (NDT). We performed a ROC curve to determine the ability of this condition to predict the thickness. Immunohistochemistry was implemented to detect macrophagocytes and endotheliocytes.

**Results:**

Based on χ2 and Spearman’s rho test, LDLC, TC, and CS are all related with intimal and media thickness (*P* < 0.05). However, in multiple linear regression, CS is still a risk factor of thickness (*P* < 0.05) but LDLC and TC are not. High levels of LDLC, TC, and CS were correlated with poor NDT (*P* < 0.05). This condition can predict the thickness sensitively. The endarterium is richest in macrophagocytes, and the arrangement of endotheliocytes is disordered and cracked under CS.

**Conclusion:**

CS is the main independent risk factor for AS and intimal (and media) thickness, rather than LDLC or TC.

## Background

The incidence and prevalence of cerebrovascular disease in both urban and rural areas of the world are far higher than other diseases, seriously affecting quality of life and increasing individual and societal burdens [1]. Ischemic cerebrovascular disease (ICVD) accounted for 80–85% of all cerebrovascular disease and 80% of ICVD patients suffer from intracranial or outside brain artery lesions. Among these lesions, large arterial disorder (LAD) can lead to various forms of ischemic events and easily cause a severe ischemic stroke (IS) [2, 3]. The main cause of LAD is atherosclerosis (AS), in which the changes of vascular intimal and media thickness play a critical role. However, at present the occurrence and recurrence of ICVD cannot be effectively controlled. One primary reason for this problem is that the inducing factor of thickness changes within AS is still unclear, making it difficult to achieve timely discovery, dynamic monitoring, and effective control of atherosclerotic stenosis and vulnerable plaque. Meeting these challenges dominates ICVD research [4].

The atherosclerosis underlying large arterial disorder can also cause ICVD, whose pathogenic mechanisms mainly include two features [5]. The first is that atherosclerotic plaques cause cerebral artery stenosis or occlusion, leading to insufficient cerebral perfusion in the distal brain. The second is that the deciduous atherosclerotic plaque directly embolizes the distal cerebral artery. Atherosclerosis is a chronic inflammatory disease, and the traditional risk factors of cerebrovascular disease, such as hyperlipidemia (the concentration of low-density lipoprotein cholesterol and total cholesterol exceeding the normal level), can only explain the pathophysiological mechanism of about 40–50% patients with atherosclerosis [6]. One could say that hyperlipidemia must be the leading risk factor for changed vascular intimal and media thickness in the process of atherosclerosis. However, about 40% of atherosclerotic patients without traditional risk factors are associated with chronic stress (CS) [7].

CS refers to the non-specific systemic reaction that occurs when the body is stimulated by various internal and external environmental factors for a long time. From an etiological perspective, the cause of the disease is often abnormal social psychological factors, which can lead to biological changes in the body and finally morphological changes of the corresponding organ [8]. Clinical epidemiological studies have shown that CS plays an important role in atherosclerotic diseases, but the high number of confounding factors in clinical surveys has prevented the elucidation of this role [9]. Accordingly, it is particularly important to establish appropriate animal models of CS and atherosclerosis to explore their interaction, and to compare CS with the contributions of low density lipoprotein cholesterol (LDLC) and total cholesterol (TC).

Rabbits’ characteristics, such as the lack of a low density lipoprotein receptor to form a hyperlipidemia model easily, moderate volume, and good response to a high cholesterol diet, make them the most widely used species for this type of research [10]. Therefore, this study employs New Zealand rabbits to research the role of CS in the development of AS and to compare it with the effects of LDLC and TC. The research starts by examining chronic stress, which is an abnormal social psychological factor. We establish the animal model of CS and atherosclerosis, observe and analyze the effect of chronic stress on vascular intimal and media thickness in the progress of atherosclerosis, and finally compare it with the LDLC and TC results, in order to provide a theoretical basis for the prevention of atherosclerotic occurrence and development.

## Methods

### Animals and diet

Thirty-seven New Zealand white rabbits (3.0 months old) weighing 3.0 ± 0.2 kg were acquired from the Institute of Laboratory Animal Sciences, Chinese Academy of Medical Sciences (CAMS), & Peking Union Medical College. The rabbits were raised in three groups randomly and they adapted to the research environment for 1 week. All rabbits were raised in independent cages (60 cm × 60 cm × 60 cm) and could obtain water and food freely in an appropriate environment: a temperature of 25 ± 2 °C; a relative humidity of 60% ± 2%; and a 12-h dark/light cycle, with lights on at 08:00. The experimental and ethical conditions was authorized by the Animal Care and Use Committee and every effort was made to minimize suffering and reduce pain during the procedures.

Beijing Keao Third-Feed Co provided the diet. After 1 week of acclimation, the rabbits were divided into three groups randomly: 1) the normal group (*n* = 11), fed with normal chow composed of 4.2% mono-unsaturated fat, 4.0% saturated fat, 45% carbohydrates, 2.0% polyunsaturated fat, and a total of 11.4% of kcal from fat; 2) the high-fat diet (HF) group (*n* = 13), fed with 91.23% basic feed and 2.2% cholesterol, 2.5% sugar, 5.2% lard, 0.3% propylthiouracil and 0.36% cholate; and 3) the high-fat diet plus chronic stress (HF + CS) group (*n* = 13), fed with 91.23% basic feed and 2.2% cholesterol, 2.5% sugar, 5.2% lard, 0.3% propylthiouracil, and 0.36% cholate under chronic stress conditions for 8 weeks.

### Chronic stress (CS) assay

The CS procedure used in the experiment is a slightly modified variation of the scheme previously described [11]. First, noise stimulation: 5 s of alarm, at about 110 dB (dB), then 5 min of silence for 3 h. Second, flash and alarm stimulation: a flash plus a continuous alarm (about 85 dB) for 2 h after lights out in the animal house. Third, constraint stimulation: limiting the rabbit’s activity with a rabbit box for 3 h. Fourth, lighting stimulation: after 7 pm, the rabbits in the CS group were still stimulated by the lighting. Fifth, fear stimulation: the cages are hung on hooks so they incline when the rabbits move slightly. This condition continues for 2 h. Sixth, no water for 24 h. Seventh, no food for 24 h. The chronic stress period is 7 days, and there is one cycle in each month. The procedure continues for 2 months.

### Biochemical analysis assay

Blood samples were collected under chloral hydrate anesthesia and centrifuged at 3500 rpm for 15 min by a refrigerated centrifuge (HEMA, Britain), then the serum was transferred into a separate vial and stored at 4 °C. Serum concentrations of glutamic oxaloacetic transaminase (AST), low-density lipoprotein cholesterol (LDLC), total cholesterol (TC), and triglyceride (TG) were measured by enzymatic assays using an automated biochemical analyzer (Beckman, USA).

### Histology assay of abdominal aorta

After blood collection, the abdominal aorta was dissected and cut into parts. A part of the aorta was fixed in a 4% poly-formaldehyde solution and processed for paraffin embedding and sectioning. Each aorta was cut into 6-μm-thick transverse sections and stained with hematoxylin-eosin (HE) after being fixed in a 4% poly-formaldehyde solution embedded in paraffin wax. The measurements of intima thickness and media thickness were made in HE stained sections of abdominal aortae under a microscope (Axio Zoom V16, ZEISS, Germany), using Image Pro Plus 5.0 software.

### Immunohistochemistry to detect macrophagocyte and vascular endothelial cells

All tissue samples obtained were taken apart to make paraffin sections. The paraffin sections are deparaffinized to water, sealed with H_2_O_2_, and washed with double-distilled water. After antigen retrieval, immunohistochemistry was used to detect the macrophagocyte and vascular endothelial cells. The specific detection steps are performed according to the instructions of the VECTASTAIN® Elite® ABC Kit (Vector Laboratories, USA), as follows: antigen-fixed paraffin sections were washed with PBS 2–3 times (5 min/times), then blocked with 10% goat serum (TransGen Biotech, China) at 37 degrees for 20 min. Filter paper was used to remove the serum, then YAP or TAZ Rabbit polyclonal antibody (Abcam, UK) was added dropwise and incubated overnight at 4 °C, washed three times with PBS (5 min), and incubated with Goat Anti-Rabbit monoclonal antibody at 37 °C for 1 h. We used DAB for color development (PBS instead of primary antibody was used as a negative control). Each paraffin section was photographed at 5 fields and counted. An Anti-RAM11 monoclonal antibody was used to detect macrophagocytes, and an Anti-CD31 monoclonal antibody was used to detect the vascular endothelial cells.

### Statistical analysis

The results are expressed as the mean ± SEM. All statistical analyses were conducted using SPSS software (version 21.0). Statistical significance was determined by a Student’s t-test when two groups were compared, or by ANOVA and post-hoc two-tailed Newman-Keuls test when three groups were compared. Non-depression time (NDT) was defined as the time from the date of implementing intervention to the date when the rabbit’s inactivity behavior increased and the locomotor behavior decreased. Rabbits which were non-depressive at last observation were censored for Cox proportional regression analysis. Associations between intimal and media thickness and potential related factors were analyzed using the χ2 test, while univariate analysis for potential prognostic factors for NDT were conducted using Cox proportional hazards regression analysis. We performed a receiver operator characteristic curve analysis to determine the ability of the condition (normal, high-fat diet, or high-fat diet plus chronic stress) to predict intimal and media thickness. For correlation analysis, the Spearman-rho test was used to compare the histological, biochemical, social, and general variables. Any test results reaching a liberal statistical threshold of *p* < 0.2 for each comparison were then entered into a multivariable linear regression model, to identify independently predictive factors for intimal and media thickness. The risk factors were entered into the same model. Then, the intimal and media thickness values were converted into natural logarithmic equivalent values for statistical analysis. Variance inflation factors were calculated to quantify the severity of multicollinearity in the multivariate linear regression model. We conducted a histogram and Shapiro Wilk test to determine the residual distribution, and we found the residuals had a well-modeled normal distribution. A Wilcoxon signed-rank test was performed to compare the intimal and media thickness. A *p* value of less than 0.05 was considered statistically significant.

## Results

### Rabbit characteristics

Rabbits’ characteristics are shown in Table 1. The number of females is roughly the same as the males (19:18), and the conditions were normal environment, high-fat diet, or high-fat diet plus chronic stress.

**Table 1:** Clinicopathological variables and the status of intimal and media thickness

Characteristics			Intimal thickness		*P*	Media thickness		*P*
			Low (%)	High (%)		Low (%)	High (%)	
Sex	Female	19	8 (21.6%)	11 (29.7%)	0.842	13 (35.1%)	6 (16.2%)	0.642
	Male	18	7 (18.9%)	11 (29.7%)		11 (29.7%)	7 (18.9%)	
Condition*	normal	11	11 (29.7%)	0 (0.0%)	< 0.001	11 (29.7%)	0 (0.0%)	< 0.001
	HF	13	4 (10.8%)	9 (24.3%)		10 (27.0%)	3 (8.1%)	
	HF + CS	13	0 (0.0%)	13 (35.1%)		3 (8.1%)	10 (27.0%)	
AST*	Low	19	12 (32.4%)	7 (18.9%)	0.004	13 (35.1%)	6 (16.2%)	0.642
	High	18	3 (8.1%)	15 (40.5%)		11 (29.7%)	7 (18.9%)	
LDLC*	Low	13	11 (29.7%)	2 (5.4%)	< 0.001	12 (32.4%)	1 (2.7%)	0.010
	High	24	4 (10.8%)	20 (54.1%)		12 (32.4%)	12 (32.4%)	
TC*	Low	12	11 (29.7%)	1 (2.7%)	< 0.001	11 (29.7%)	1 (2.7%)	0.018
	High	25	4 (10.8%)	21 (56.8%)		13 (35.1%)	12 (32.4%)	
TG	Low	25	12 (32.4%)	13 (35.1%)	0.182	17 (45.9%)	8 (21.6%)	0.564
	High	12	3 (8.1%)	9 (24.3%)		7 (18.9%)	5 (13.5%)	

Pearson’s chi-squared test was used. *P < 0.05

### Associations between intimal and media thickness and potential related factors

Associations between the intimal and media thickness of the abdominal aorta and potential related factors are summarized in Table 1. Twenty two of the thirty-seven rabbits showed very high levels for intimal thickness and 15 showed very low levels for intimal thickness; 15 of the 37 rabbits showed very high levels for media thickness and 24 showed very low levels for media thickness. Examples of intimal and media thickness in the abdominal aortae are shown in Fig. 3(a). 29.7% (11/37) of the individuals were in the normal condition, 35.1% (13/37) in the high-fat diet condition, and 35.1% (13/37) in the high-fat plus chronic stress condition. Both intimal and media thickness were significantly associated with condition (*p* < 0.001), LDLC (*p* < 0.05), and TC (*p* < 0.05). What’s more, the intimal thickness was significantly associated with AST (*p* < 0.01). No significant associations were found between intimal and media thickness and other potentially related factors. There was a significant positive association between intimal and media thickness (*p* = 0.003), as shown in Table 2.

**Table 2:** The corelationship of the expression between intimal and media thickness

Characteristics			Media thickness		p(spearman)
			Low (%)	High (%)	
Intimal thickness *	Low	15	14 (37.8%)	1 (2.7%)	0.003*
	High	22	10 (27.0%)	12 (32.4%)	
		37	24	13	

Spearman-rho test was used. *P < 0.05

### High levels of AST, LDLC, and TC in individuals; HF and CS were correlated with poor NDT

Univariate Cox proportional hazard analyses for NDT are summarized in Table 3. Higher AST values were predictive of an early NDT. In addition, univariate analysis revealed that a high fat diet and chronic stress were significantly associated with early NDT (*p* < 0.05). Among the rabbits that were fed a high-fat diet or a high-fat diet plus chronic stress, univariate analysis revealed that the high-fat diet (*p* < 0.05) and high-fat diet plus chronic stress (*p* < 0.05) conditions were all significantly associated with poor NDT (*p* < 0.05).

**Table 3:** Correlative characteristics’ effect on NDT based on univariate Cox proportional regression analysis

Characteristics			NDT		
			HR	95% CI	P
Sex	Female	19	1		0.791
	Male	18	0.901	0.416–1.951	
Condition	normal	11	1		0.036
	HF	13	4.138	1.149–14.899	
	HF + CS	13	3.561	0.990–12.808	
AST	Low	19	1		0.023
	High	18	2.657	1.148–6.154	
LDLC	Low	13	1		0.041
	High	24	2.778	1.044–7.396	
TC	Low	12	1		0.030
	High	25	3.261	1.118–9.512	
TG	Low	25	1		0.116
	High	12	1.896	0.854–4.208	

*NDT* Non-depression time, *HR* hazard ratio, *95% CI* 95% confidence interval. * *P* < 0.05

### Associations between intimal (and media) thickness and subjects’ characteristics

To ensure that condition had an impact on intimal and media thickness, we performed a further analysis of intimal and media thickness and associated risk factors. Spearman’s correlation coefficient was used in the correlation analysis, and condition (ρ = 0.658, *p* < 0.001), LDLC (ρ = 0.488, *p* = 0.002), and TC (ρ = 0.498, *p* = 0.002) were significantly correlated with intimal thickness (Table 4). In the multivariate linear regression model, holding all other variables at any fixed value, the natural logarithmic intimal thickness remained associated with condition (β = 0.632, *p* < 0.001). In the correlation analysis, condition (ρ = 0.747, *p* < 0.001), AST (ρ = 0.436, *p* = 0.007), LDLC (ρ = 0.552, *p* < 0.001), and TC (ρ = 0.446, *p* = 0.006) were significantly correlated with media thickness. In the multivariate linear regression model, condition (β = 0.083, *p* = 0.004) was significantly and independently associated with media thickness. Holding other variables constant, TG (β = 0.069, *p* = 0.013) was significantly associated with natural logarithmic media thickness (Table 4).

**Table 4:** Associations between intimal (and media) thickness and subjects’ characteristics

Characteristics	Intimal thickness					Media thickness				
	Spearman’s rank correlation coefficient coefficient		Multiple linear regression			Spearman’s rank correlation coefficient		Multiple linear regression		
	ρ^a^	*p*-value	β^b^	*p*-value	VIF	ρ^a^	*p*-value	β^b^	*p*-value	VIF
Sex	0.077	0.653	−0.012	0.927	1.145	0.026	0.877	−0.039	0.089	1.145
Condition	0.658	< 0.001*	0.632	< 0.001*	4.355	0.747	< 0.001*	0.083	0.004*	4.355
AST	0.242	0.149	−0.011	0.127	1.685	0.436	0.007*	0.001	0.781	1.685
LDLC	0.488	0.002*	− 0.040	0.288	29.772	0.552	< 0.001*	− 0.002	0.778	29.772
TC	0.498	0.002*	0.011	0.510	24.568	0.446	0.006*	− 0.001	0.788	24.568
TG	0.080	0.640	0.238	0.127	1.683	0.243	0.147	0.069	0.013*	1.683

Condition: normal; High fat diet; High fat diet plus chronic stress

*AST* glutamic oxalacetic transaminase, *LDLC* Low Density Lipoprotein Cholesterol, *TC* total cholesterol, *TG* triglyceride, *VIF* variance inflation factor

^a^Spearman’s rank correlation coefficient between intimal (and media) thickness and each variable; ρ: Spearman’s correlation coefficient

^b^Multiple linear regression analysis, β: parameter estimate;

*Significant variables

### The condition (normal; HF; HF + CS) can predict intimal and media thickness sensitively and specially by ROC curve

**Fig. 1:**
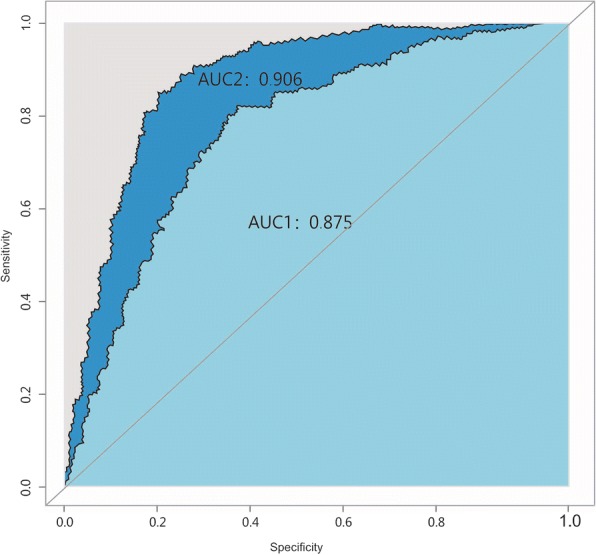
ROC curve of the predictive value of condition (normal, high-fat diet, or high-fat diet plus chronic stress) for intimal (and media) thickness. Increasing the degree of condition was predictive of intimal (and media) thickness. For the ROC curve between condition and intimal thickness, the area under the curve (AUC1) = 0.875; 95% confidence interval, 0.761–0.989; *p* < 0.001. For the ROC curve between condition and media thickness, the area under the curve (AUC2) = 0.906; 95% confidence interval, 0.805–1.000; *p* < 0.001

To identify an accurate threshold for condition predicting intimal and media thickness, we constructed receiver operator characteristic curves. Condition was associated with higher risk of intimal and media thickness (Area under the curve for intimal thickness, 0.875; 95% CI, 0.761–0.989; *p* < 0.001. Area under the curve for media thickness, 0.906; 95% CI, 0.805–1.000; *p* < 0.001). (Fig. 1).

### Histological differences between various conditions

**Fig. 2:**
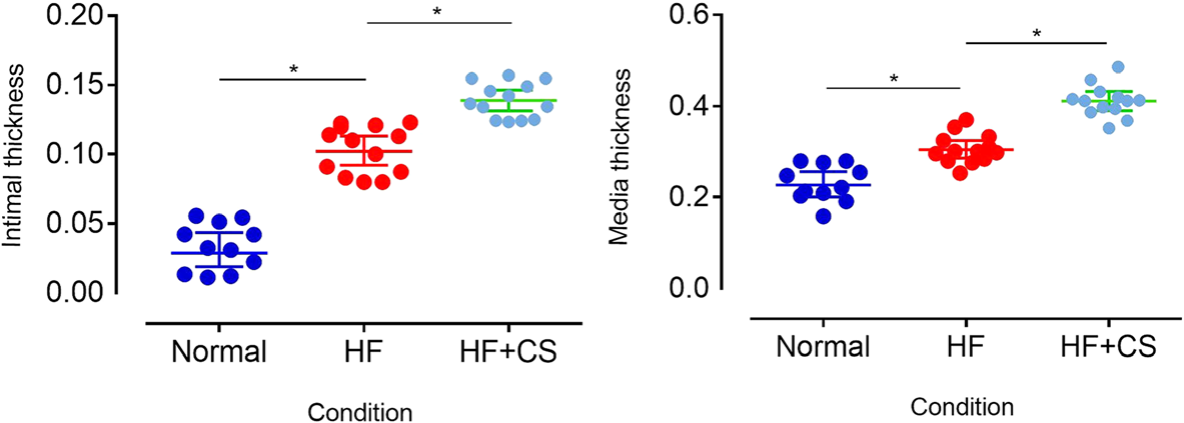
Comparative intimal (and media) thickness geometric means between the different conditions. HF: high-fat diet; HF + CS: high-fat diet plus chronic stress. *P < 0.05

**Fig. 3:**
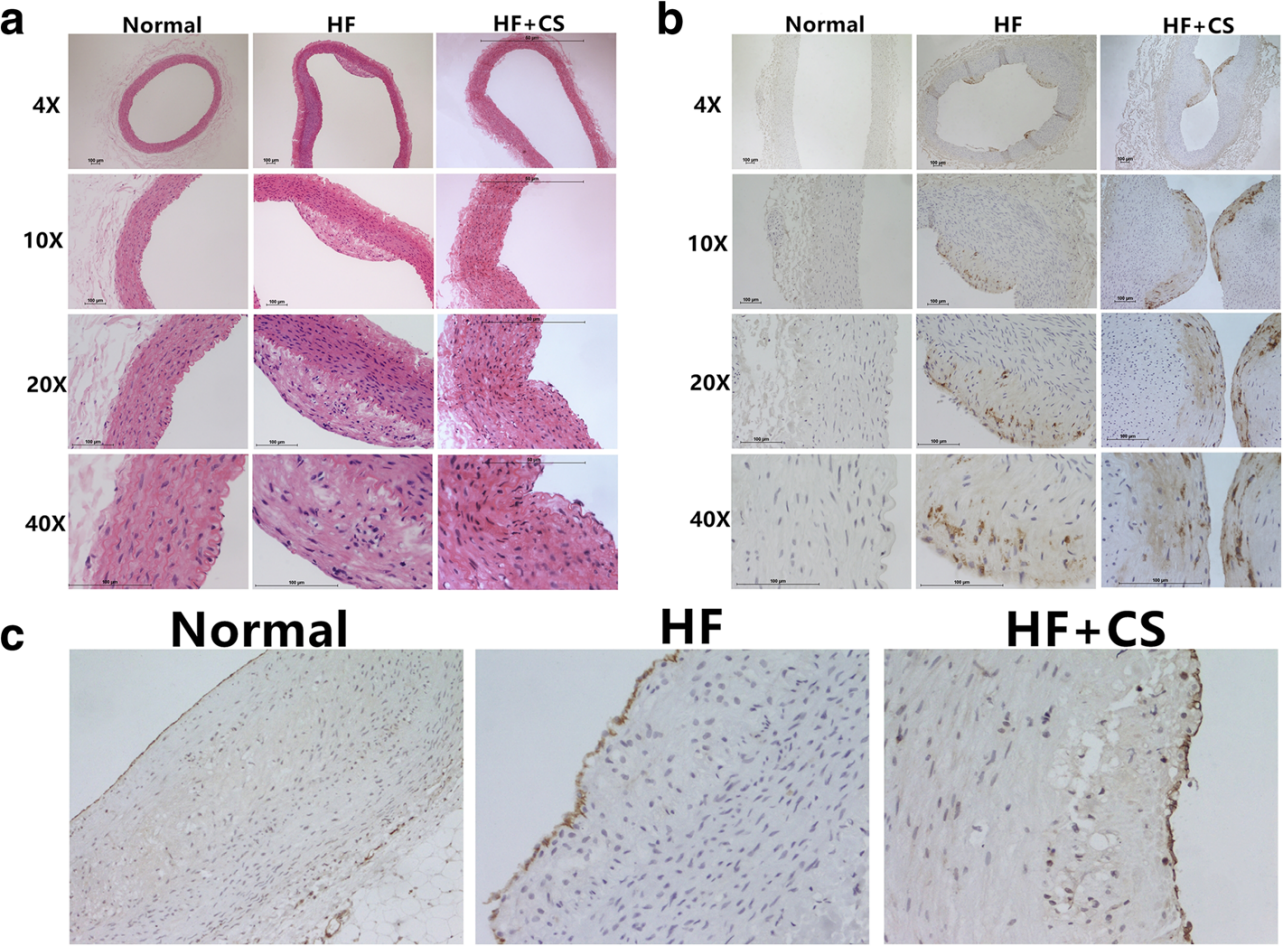
The observation of abdominal aortae by staining assay between the different conditions. **a** HE staining (4×,10×,20×,40×); **b** Immunohistochemical staining by the RAM11 marker to observe the macrophagocytes (4×,10×,20×,40×); **c** Immunohistochemical staining by the CD31 marker to observe the vascular endothelial cells. HF: high-fat diet; HF + CS: high-fat diet plus chronic stress

Through HE staining (4×,10×,20×,40×) and data analysis, we can see that the intimal and media thickness increased in this order: normal, HF, and HF + CS (Fig. 2). In the normal group the endarterium is very thin, and in the endovascular cavity there is a layer of endothelial cells and a layer of red continuous elastic membranes. The tunica media are mainly composed of smooth muscle cells, a small amount of elastic fibers, and collagen fibers. What’s more, the vascular adventitia and tunica media are divided by an external elastic membrane. In the high-fat diet group, the abdominal aorta plaque is mainly type II, but there are a few type III lesions, as well as some local thickening of the intima, intracellular lipid accumulation, and the formation of foam cells. In the high-fat diet plus chronic stress group, the tunica media were thickened and the number of smooth muscle cells increased, and they were disordered with morphological changes. Specifically, some local hyperplasia was prominent in the lumen, as shown in Fig. 3(a). Through immunohistochemical staining, we can see that the endarterium is rich in macrophagocytes in the HF group compared with the normal group, but there are more in the HF + CS group (Fig. 3(b)). Furthermore CD31-markered staining shows that the arrangement of endothelial cells is regular in the normal group, disordered in the HF group, and disordered and cracked in the HF + CS group (Fig. 3(c)).

## Discussion

Cerebrovascular disease, often driven by atherosclerosis, accounts for the highest percentage of disability and death among both urban and rural populations. It also brings about the highest medical burden, making it a major public health problem and creating a serious economic and mental burden for patients’ families and societies. What’s more, the mortality rate of patients with cerebrovascular disease is rising at an annual rate of 1%. In recent years, with the rapid development of the economy and the increased aging population, the incidence of chronic non-communicable diseases has grown higher and higher, especially in terms of diabetes, high blood pressure, hyperlipidemia, hyperuricemia, obesity, and chronic psychological stress. Stress can specifically lead to the occurrence of atherosclerosis, and the problem is becoming more and more serious [12, 13]. In the process of atherosclerosis, there are changes in vascular intima and media thickness. Then, the plaque can be built up and cause a hemal stricture, and eventually ischemic cerebrovascular disease events. Therefore, it is crucial to find the main inducing factors within the pathophysiological process of AS, to prevent the occurrence and recurrence rate of cerebrovascular diseases.

The relationship between chronic stress and intimal (and media) thickness was not clear in previous scholarship [14, 15]. ZM Yu and her colleagues [11] found that chronic stress may promote the apoptosis of smooth muscle cells and lead to thinner media thickness in the blood vessels. In their animal experiments, vascular walls with the AS lesion were dilated. Compensatory dilation of blood vessels can accommodate the growing plaques in order to maintain the bloodstream and delay the phenomenon of ischemia. In the development of AS, the external elastic membrane of the vessel continues to expand outward to accommodate the plaque and maintain the shape of the vascular lumen. When the plaque area is greater than 40%, the lumen is narrowed. The change in vascular wall structure is due to the remodeling of blood vessels, and smooth muscle cells (SMCs) play an important role in the structure and function of the vascular wall. It was also observed that the tunica media in the blood vessel were significantly thickened, but the media’s elastic fiber layer decreased in the atherosclerotic blood vessel after chronic stress.

Dyslipidemia is the first recognized independent risk factor for AS and intimal (and media) thickness. Low-density lipoprotein is a cholesterol-rich lipoprotein. Among the many risk factors, elevated serum LDLC and TC levels are the only precipitators that can induce and promote atherosclerosis without other synergistic risk factors [16]. Low-density lipoprotein cholesterol is the main pathogenic factor of atherosclerosis and its oxidative modification product, the oxidized low-density lipoprotein (ox-LDL), which can snap to mononuclear cells and be recognized and absorbed by monocyte-macrophage scavenger receptors. As a result, foam cell and fatty plaques will form. Furthermore, Bożena Króliczewska [17] noted that dietary fat is considered one of the most important factors associated with blood lipid metabolism and plays a significant role in the cause and prevention of atherosclerosis, which has been widely accepted as an inflammatory disease of the vascular system.

However, we find that by using Univariate Cox proportional hazard analyses, high levels of AST, LDLC, and TC in individuals, along with HF and CS, were correlated with non-depression time. In the Spearman’s correlation coefficient and χ2 test analysis, condition (normal, high-fat diet, and high-fat diet plus chronic stress), LDLC and TC were significantly related with intimal (and media) thickness. But in the multivariate linear regression model, holding all other variables at any fixed value, natural logarithmic intimal thickness remained associated with condition. By an ROC curve, the condition can predict intimal and media thickness sensitively and specially. In a comprehensive analysis, the levels of LDLC and TC may not be the leading reasons for the change of intimal (and media) thickness compared with chronic stress. This is similar to Qi Yu’s finding that dietary restriction affected glucose and lipid metabolism but did not ameliorate atherosclerosis in rabbits, when associated with lipid lowering via dietary cholesterol withdrawal [18].

Based on our histologic analysis, chronic stress can make macrophages gather at the endangium, and AS is a chronic inflammatory disease [19]. Inflammation response, a key factor that makes plaque unstable, exists throughout the AS pathological process [20]. One of its mechanisms is that macrophages secrete matrix metalloproteinases (MMPs), which can degrade the collagen fibrous cap and extracellular matrix components. Chronic stress causes hemodynamic changes, resulting in shear force on the vascular wall and localized endothelial injury. Chronic stress can also lead to imbalances between nitric oxide (NO) and endothelin (ET), and to endothelial dysfunction. Vascular endothelial cells are arranged in a single longitudinal layer in the lining of blood vessels. Damage to a vascular endothelial cell is the initial factor of the pathophysiological process and development of AS. At the same time, it is the target treatment point for preventing cerebrovascular disease. Vascular endothelial cells not only have a barrier function and endocrine and immune activity, but also play an critical role in the development of atherosclerotic pathophysiology [21]. This includes involvement in vasoconstriction and vasodilation, which can control blood pressure, coagulation, fibrinolysis, atherosclerosis, angiogenesis, and material exchange. For example, it can lead to the high expression of adhesion molecules in inflammation, which can interact with the adhesion molecules of white blood cells in the blood flow, thus mediating white blood cells to pass through the lining of blood vessels and enter into the subendothelial tissue to promote the formation of the AS plaque [22, 23]. Therefore, damaged vascular endothelial cells can expose the damaged vascular intima to a variety of pathogenic factors, activating the intrinsic and extrinsic coagulation pathways. Local thrombosis can lead to early luminal stenosis or occlusion. Simultaneously, the local damaged blood vessels can produce a variety of cytokines, chemokines, and inflammatory mediators, such as platelet-derived growth factor, insulin-like growth factor, etc. [24, 25]. Under the stimulus of these factors, a massive proliferation of smooth muscle cells causes neointimal hyperplasia and aggravates the pathological outcomes of vascular stenosis, which mainly promotes the pathophysiological process of AS. Research has found that vascular endothelial cell injury is one of the main original factors of AS. The pathophysiological development of AS, caused by chronic stress, begins with damage to the vascular endothelial cell. At present we can’t effectively control the pathophysiological process of AS, so we can’t reduce the occurrence and recurrence rate of cerebrovascular diseases [26]. One of the likely reasons for this failure is that we can’t achieve the goal of dynamically monitoring vascular endothelial cell injuries caused by chronic stress.

## Conclusions

Chronic stress is an independent risk factor for AS and intimal (and media) thickness. The levels of LDLC and TC may not be the leading reason for thickness changes compared with the effect of chronic stress. In this study, the experiment condition (normal, HF, or HF + CS) predicted intimal and media thickness sensitively and specially. Accordingly, one potential mechanism is that chronic stress can make macrophages gather at the endangium and accelerate damage to the lining of vascular endothelial cells.

## Abbreviations

AS: Atherosclerosis
AST: Glutamic oxalacetic transaminase
CAMS: Chinese Academy of Medical Sciences
CS: Chronic stress
ET: Endothelin
HE: Hematoxylin-eosin
HF: High fat diet
ICVD: Ischemic cerebrovascular disease
IS: Ischemic stroke
LAD: Large arterial disorder
LDLC: Low density lipoprotein cholesterol
MMPs: Matrix metalloproteinases
NDT: Non-depression time
NO: Nitric oxide
ox-LDL: The oxidized low density lipoprotein
SMCs: Smooth muscle cells
TC: Total cholesterol
TG: Triglyceride
